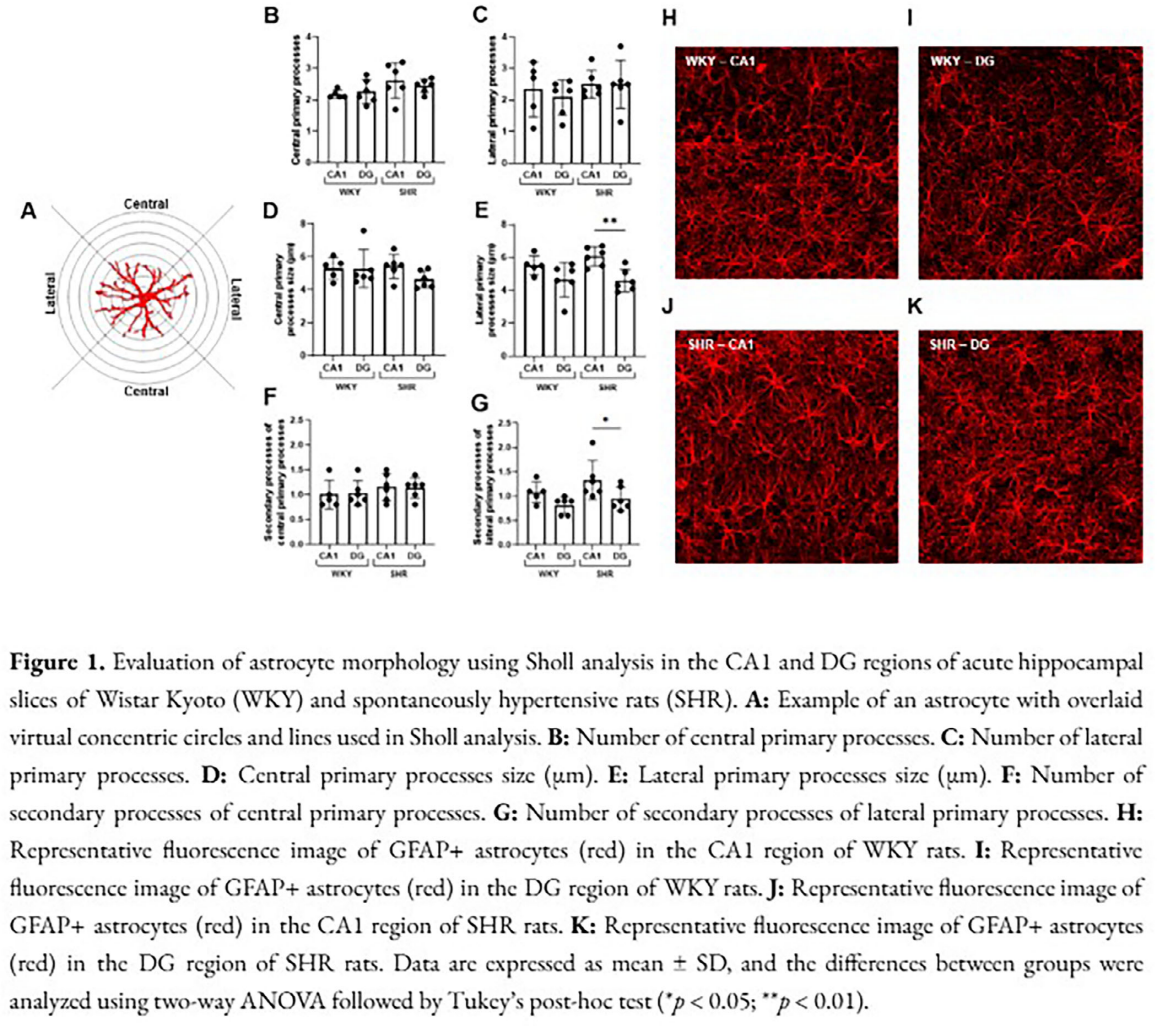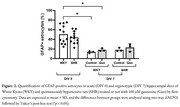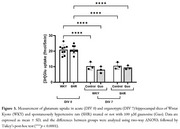# Impact of Systemic Arterial Hypertension on Astrocytic Morphology and Function in Acute and Organotypic Hippocampal Slices

**DOI:** 10.1002/alz70855_104937

**Published:** 2025-12-24

**Authors:** Felippo Bifi, Francieli Rohden, Leo Martins, Débora Guerini de Souza, Eduardo R. Zimmer, Diogo O. Souza

**Affiliations:** ^1^ Federal University of Rio Grande do Sul (UFRGS), Porto Alegre, RS, Brazil; ^2^ Brain Institute of Rio Grande Do Sul, PUCRS, Porto Alegre, RS, Brazil; ^3^ McGill Centre for Studies in Aging, Montreal, QC, Canada

## Abstract

**Background:**

Systemic arterial hypertension (SAH), characterized by persistently elevated blood pressure, is among the most prevalent chronic diseases globally. It is a well‐established risk factor for cognitive decline, as it impacts the morphology of brain cells, potentially leading to Alzheimer's disease. This study explored the morphological and functional cellular alterations in the hippocampus of spontaneously hypertensive rats (SHR) using both acute and organotypic slice cultures, treated with guanosine, an endogenous nucleoside that may exert its effects through the modulation of the glutamatergic system in astrocytes.

**Method:**

We utilized 5–6‐month‐old male SHR and Wistar Kyoto (WKY) rats to prepare 150 µm hippocampal slices, including acute slices (day‐*in‐vitro* [DIV] 0) and organotypic slices (DIV 7). These slices were treated with 100 µM guanosine throughout the culture period. Immunofluorescence staining with glial fibrillary acidic protein (GFAP) was conducted to analyze the primary and secondary processes of astrocytes in the cornu ammonis 1 (CA1) and dentate gyrus (DG) regions using a modified Sholl analysis. GFAP labeling was also assessed via flow cytometry to characterize astrocytic cell profiles. Additionally, glutamate uptake was measured in the hippocampal slices.

**Result:**

In acute slices, we observed a decrease in the lateral primary processes size [*p* <0.01] and fewer secondary processes branching from lateral primary processes [*p* <0.05] in a hippocampal region‐dependent manner noted exclusively in SHR animals (Figure 1). Additionally, a significant reduction in the number of GFAP+ astrocytes was detected in organotypic slices of WKY rats [Control and Guanosine: *p* <0.05], with no detectable effect of guanosine treatment (Figure 2). Furthermore, both SHR [Control and Guanosine: *p* <0.0001] and WKY [Control and Guanosine: *p* <0.0001] rats demonstrated reduced glutamate uptake in organotypic slices compared to acute slices, irrespective of guanosine treatment (Figure 3).

**Conclusion:**

The findings highlight SHR‐specific astrocytic morphological changes in acute slices, coupled with reduced astrocyte numbers and impaired glutamate uptake in organotypic cultures from both WKY and SHR rats. These alterations may contribute to the cognitive decline associated with SAH, underscoring the need for further research to elucidate the role of astrocytic dysfunction in SAH and its potential link to Alzheimer's disease.